# The Prevalence of Post-traumatic Stress Disorder Among Health Care Workers During the COVID-19 Pandemic: An Umbrella Review and Meta-Analysis

**DOI:** 10.3389/fpsyt.2021.764738

**Published:** 2021-11-15

**Authors:** Ali Sahebi, Atefeh Yousefi, Kamel Abdi, Yousef Jamshidbeigi, Siamak Moayedi, Mercedes Torres, Ulrich Wesemann, Hojjat Sheikhbardsiri, Mohamad Golitaleb

**Affiliations:** ^1^Non-communicable Diseases Research Center, Ilam University of Medical Sciences, Ilam, Iran; ^2^Neurology Resident, Department of Neurology, Shohadaye Tajrish Hospital, Shahid Beheshti University of Medical Science, Tehran, Iran; ^3^Nursing Department, Faculty of Medicine, Komar University of Science and Technology, Sulaymaniyah, Iraq; ^4^Department of Anesthesia, School of Paramedical, Ilam University of Medical Sciences, Ilam, Iran; ^5^Department of Emergency Medicine, University of Maryland School of Medicine, Baltimore, MD, United States; ^6^Department of Psychiatry, Psychotherapy and Psychotraumatology, Bundeswehr Hospital, Berlin, Germany; ^7^Health in Disasters and Emergencies Research Center, Institute for Futures Studies in Health, Kerman University of Medical Sciences, Kerman, Iran; ^8^Department of Nursing, School of Nursing, Arak University of Medical Sciences, Arak, Iran

**Keywords:** COVID-19, PTSD, stress disorder, Health Care Provider, HCWs

## Abstract

**Introduction:** Frontline health care workers (HCWs) have had an increased risk of developing health problems during the COVID-19 pandemic. In addition to physical illness, they have experienced mental health challenges, including post-traumatic stress disorder (PTSD). The aim of this study is to investigate the prevalence of PTSD among HCWs during the COVID-19 pandemic via an umbrella review and meta-analysis.

**Methods:** This study was conducted using the Preferred Reporting Items for Systematic Reviews and Meta-Analyses (PRISMA) guideline to perform a systematic literature search using various medical databases (Web of science, PubMed, Scopus, Cochrane, ProQuest, Science Direct, Embase, and Google scholar). The search included all articles published through the first of January 2020 the end of March 2021. The systematic review and meta-analysis studies that reported the prevalence of PTSD among health care workers were included in the study, and studies that reported the prevalence of PTSD in normal people or other epidemics were excluded. The random effects model was used to perform a meta-analysis, and the *I*^2^ index was used to evaluate heterogeneity among studies. Publication bias was assessed using the Egger test. Data was analyzed using STATA (version 14) software.

**Results:** The initial literature search yielded 145 studies. After excluding duplicates and assessing the quality of the studies, 7 studies were selected for meta-analysis. The results showed that the overall prevalence of PTSD among HCWs during the COVID-19 pandemic was 13.52% (95% CI: 9.06–17.98, *I*^2^ = 65.5%, *p* = 0.008).

**Conclusion:** There is a high prevalence of PTSD among frontline HCWs during the COVID-19 pandemic. It is important to invest in efforts to screen HCWs for mental health disorders such as PTSD and provide them with mental health support.

## Introduction

The COVID-19 pandemic has affected both the physical and mental health of frontline health workers (HCWs). Mental health problems have been reported among these HCWs irrespective of any history of psychologic illness in the past (1–3). One meta-analysis conducted in Asian countries during the COVID-19 crisis showed that more than 20% of people developed anxiety and depression (4). Evidence suggests that the symptoms of anxiety and depression (16–28%) and self-reported stress (8%), are common psychological reactions to COVID-19 infection (5–7). Studies have also shown an increase in the incidence of feelings of anger, fear-avoidance behaviors, and symptoms of post-traumatic stress disorder (PTSD) (8, 9).

Throughout the COVID-19 pandemic, HCWs have played a vital role by providing direct care for some of the most severely affected patients (10). They are continually exposed to physical and psychological stressors associated with this work (11). As a result, many have reported increased feelings of irritability, anger, depressed mood, and emotional instability. These individuals are at a higher risk for developing PTSD when compared with the general population (12, 13). This risk is partly attributable to a work environment where many HCWs lack appropriate supplies of personal protective equipment. Furthermore, they bear witness to the ongoing suffering and death of many patients (14). One Chinese study showed that the prevalence of PTSD among HCWs during the COVID-19 crisis was about 9.8%. Those most likely to experience PTSD in this study were nurses who worked on the frontlines and expressed a lack of confidence in protective measures (15, 16).

Numerous systematic reviews and meta-analyses have been conducted to assess for PTSD among HCWs during the COVID-19 pandemic. However, there is no comprehensive study to pool these results and report the overall prevalence of PTSD. This study is the first umbrella review that has evaluated the prevalence of PTSD among healthcare workers globally. Umbrella studies are rare and are among the most comprehensive studies and have the highest level at the pyramid of medical evidence. The aim of this study was to estimate the overall prevalence of PTSD among HCWs by conducting an umbrella review and meta-analysis.

## Methods

The Preferred Reporting Items for Systematic Reviews and Meta-Analyses (PRISMA) guideline was used to conduct this study (17). The study protocol was registered in the International Prospective Register of Systematic Reviews (PROSPERO) under the code of CRD42021248943.

### Databases and Search Strategy

PubMed, Scopus, Cochrane, ProQuest, Science Direct, Embase, and Google Scholar, and Web of Science were searched to identify relevant studies. The initial search strategy was developed for PubMed and then adjusted to meet the requirements of the other databases. The searches in databases were performed from January 1, 2020 to March 31, 2021. In order to avoid limiting the search results, time limitation wasn't applied in the search strategy, and after identifying the search results, time was applied through filtering. PTSD is a disorder, according to ICD-10, characterized by (1) flashbacks or nightmares about the traumatic event which produce terror and strong physiological reactions, (2) avoidance of memories or thoughts related to the event, or to avoid activities, situations or persons related to, and (3) a lasting perception of a current noticeable threat (12, 13). The search strategy in the database was as follows: “posttraumatic stress disorder,” “posttraumatic neuroses,” “Post Traumatic Stress Disorder^*^,” “chronic post-traumatic stress disorder,” “delayed-onset post-traumatic stress disorder,” “acute post-traumatic stress disorder,” PTSD, “Mental health disorder^*^,” “2019 novel coronavirus disease,” COVID19, “COVID-19 pandemic,” “SARS-CoV-2 infection,” “COVID-19 virus disease,” “2019 novel coronavirus infection,” “2019-nCoV infection,” “Coronavirus disease 2019,” “2019-nCoV disease,” “COVID-19 virus infection,” “Health Personnel,” “Health Care Provider^*^,” “Health worker^*^,” “Healthcare Provider^*^,” “Healthcare Worker^*^,” “Health care professional^*^,” “medical staff,” “Medical worker^*^,” “Systematic review,” “meta-analysis,” “meta-analytic.” Search strategies for different databases are referenced in Table 1.

**Table 1:** Search strategies for various databases.

**Database**	**Search strategy**	**Number**
Pubmed	[(“posttraumatic stress disorder” OR “posttraumatic neuroses” OR “Post Traumatic Stress Disorder*” OR “chronic post-traumatic stress disorder” OR “delayed-onset post-traumatic stress disorder” OR “acute post-traumatic stress disorder” OR PTSD OR “Mental health disorder*”) AND (“2019 novel coronavirus disease” OR COVID19 OR “COVID-19 pandemic” OR “SARS-CoV-2 infection” OR “COVID-19 virus disease” OR “2019 novel coronavirus infection” OR “2019-nCoV infection” OR “Coronavirus disease 2019” OR “2019-nCoV disease” OR “COVID-19 virus infection”) AND (“Health Personnel” OR “Health Care Provider*” OR “Health worker*” OR “Healthcare Provider*” OR “Healthcare Worker*” OR “Health care professional*” OR “medical staff” OR “Medical worker*”) AND (“Systematic review”) AND (“meta-analysis”[tiab] OR “meta-analytic”)]	18
Scopus	{[ALL(“posttraumatic stress disorder”) OR ALL(“posttraumatic neuroses”) OR ALL(“Post Traumatic Stress Disorder*”) OR ALL(“chronic post-traumatic stress disorder”) OR ALL(“delayed-onset post-traumatic stress disorder”) OR ALL(“acute post-traumatic stress disorder”) OR ALL(PTSD) OR ALL(“Mental health disorder*”)] AND [ALL(“2019 novel coronavirus disease”) OR ALL(COVID19) OR ALL(“COVID-19 pandemic”) OR ALL(“SARS-CoV-2 infection”) OR ALL(“COVID-19 virus disease”) OR ALL(“2019 novel coronavirus infection”) OR ALL(“2019-nCoV infection”) OR ALL(“Coronavirus disease 2019”) OR ALL(“2019-nCoV disease”) OR ALL(“COVID-19 virus infection”)] AND [ALL(“Health Personnel”) OR ALL(“Health Care Provider*”) OR ALL(“Health worker*”) OR ALL(“Healthcare Provider*”) OR ALL(“Healthcare Worker*”) OR ALL(“Health care professional*”) OR ALL(“medical staff”) OR ALL(“Medical worker*”)] AND [ALL(“Systematic review”)] AND [TITLE-ABS(“meta-analysis”) OR ALL(“meta-analytic”)]}	138
ISI	{[TS = (“posttraumatic stress disorder”) OR TS = (“posttraumatic neuroses”) OR TS = (“Post Traumatic Stress Disorder*”) OR TS = (“chronic post-traumatic stress disorder”) OR TS = (“delayed-onset post-traumatic stress disorder”) OR TS = (“acute post-traumatic stress disorder”) OR TS = (PTSD) OR TS = (“Mental health disorder*”)] AND [TS = (“2019 novel coronavirus disease”) OR TS = (COVID19) OR TS = (“COVID-19 pandemic”) OR TS = (“SARS-CoV-2 infection”) OR TS = (“COVID-19 virus disease”) OR TS = (“2019 novel coronavirus infection”) OR TS = (“2019-nCoV infection”) OR TS = (“Coronavirus disease 2019”) OR TS = (“2019-nCoV disease”) OR TS = (“COVID-19 virus infection”)] AND [TS = (“Health Personnel”) OR TS = (“Health Care Provider*”) OR TS = (“Health worker*”) OR TS = (“Healthcare Provider*”) OR TS = (“Healthcare Worker*”) OR TS = (“Health care professional*”) OR TS = (“medical staff”) OR TS = (“Medical worker*”)] AND [TS = (“Systematic review”)] AND [Ti = (“meta-analysis”) OR TS = (“meta-analytic”)]}	5

### Eligibility Criteria

#### Inclusion Criteria

Published studies reporting the prevalence of PTSD among HCWs during the COVID-19 pandemic were included in the meta-analysis.

#### Exclusion Criteria

The studies were excluded if: Studies reporting the prevalence of PTSD among other groups or during other epidemics were excluded from the analysis.

### Study Selection

**Figure 1:**
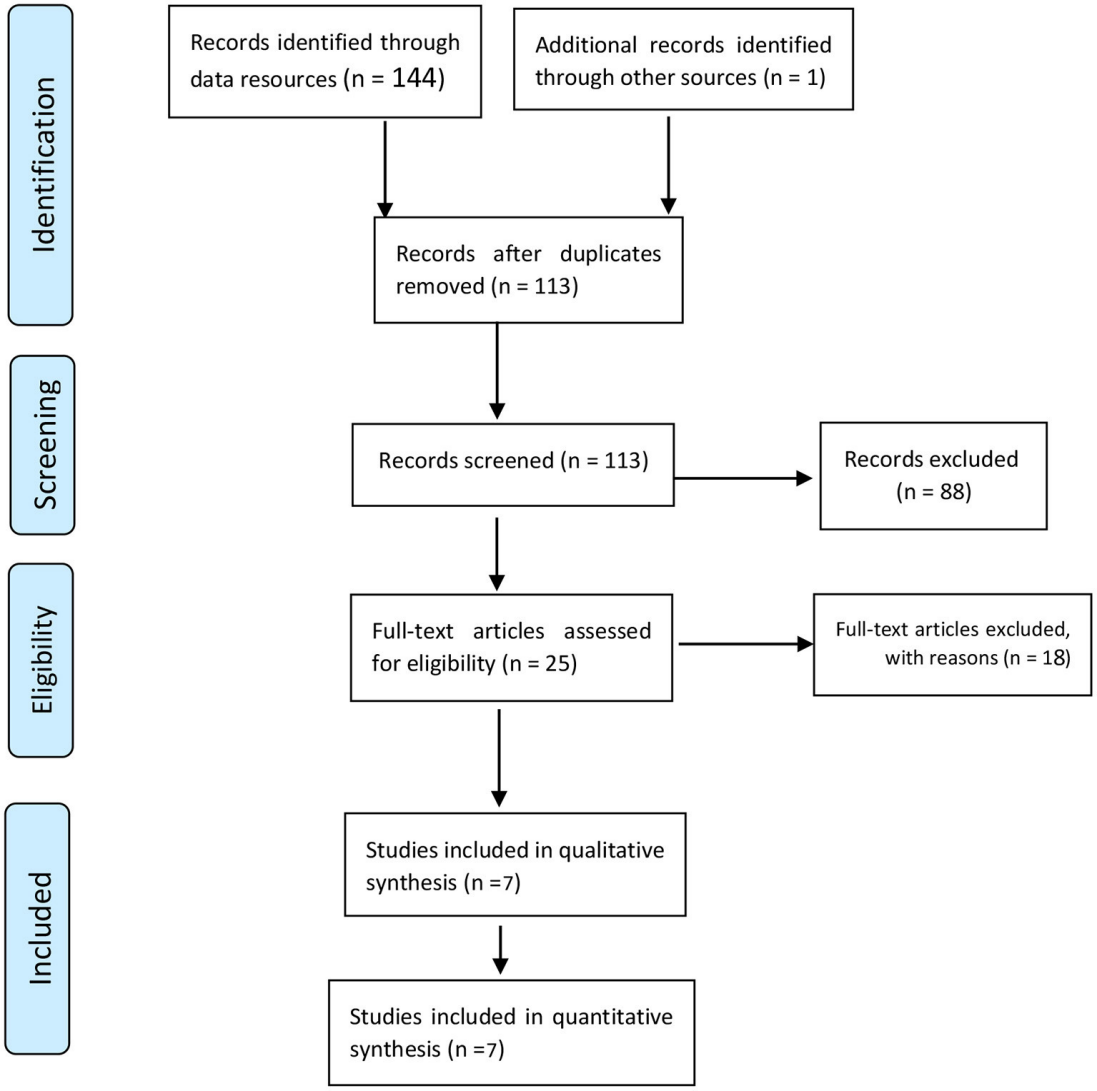
Flowchart of the selection of studies based on PRISMA.

In order to manage the search results, all of the studies identified in the initial database search were inserted into EndNote X7 software. After excluding duplicates, 113 studies were screened. Of those 113 studies, the full-texts of 25 selected studies were independently reviewed by two of the researchers (AS and MG). Ultimately, 7 studies were chosen for the quality assessment phase (Figure 1).

### Quality Assessment and Data Extraction

The methodology of the 7 selected studies was independently assessed by two of the researchers using the Assessment of Multiple Systematic Reviews v2 (AMSTAR-2) tool (18). This checklist (AMSTAR2) has 16 items and authors should answer give yes, to some extent, and no to each question. The Overall reliability of the results of this tool is classified into four levels: Critically low, low, moderate, and high. For data extraction, the two researchers (AS and MG) independently used a checklist prepared in Microsoft Word 2016 to collect the required data including the first author's name, place of study, number of participants, and rate of heterogeneity among studies included in each review.

### Statistical Analysis

The random effects model was used to perform the meta-analysis and the *I*^2^ index was used to determine heterogeneity among the studies. Each study was evaluated for its degree of heterogeneity and assigned one of the following values: <25% (none), 25–50% (moderate), 50–75% (high), and >75% (very high) (19). Publication bias in each study was assessed using the Egger‘s test. The data was analyzed using STATA (version 14) software.

## Results

**Figure 2:**
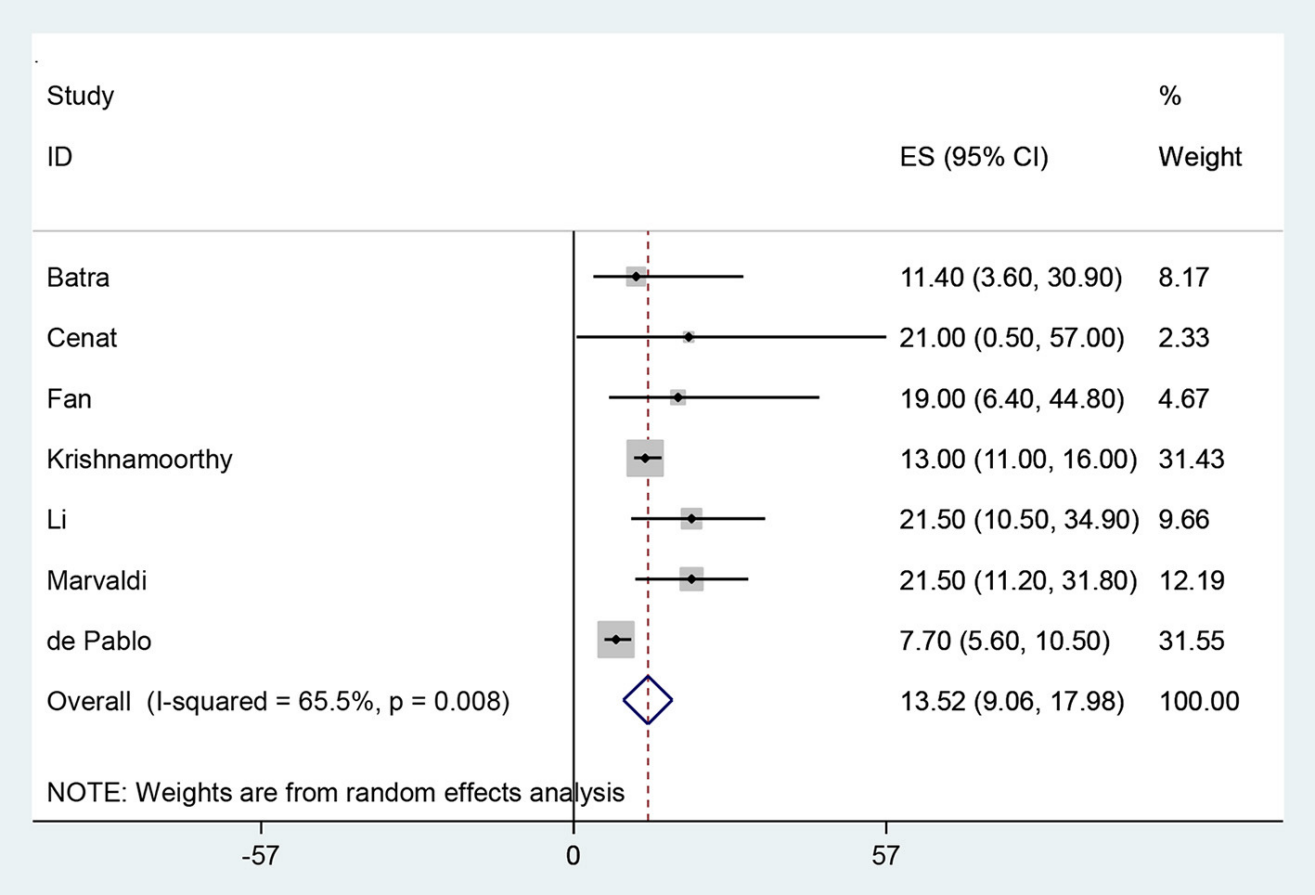
The Forest plot of overall and individual prevalence of PTSD in the studies with 95% confidence interval.

**Figure 3:**
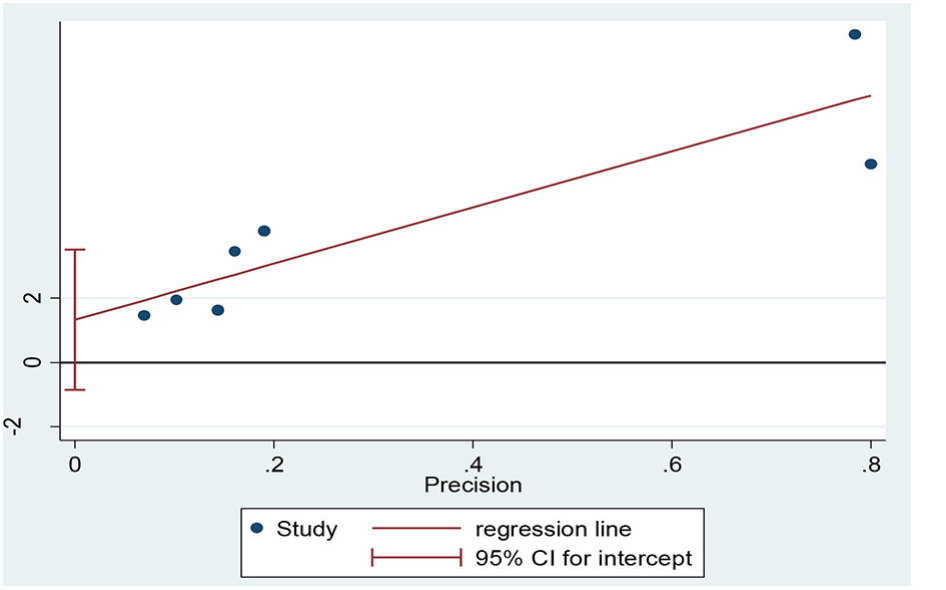
Publication bias based on Egger‘s test.

Five hundred eighty eight publications which included a total of 323,459 HCWs were included in the meta-analysis phase. Table 2 shows the specifications and data of each study. The overall prevalence of PTSD among HCWs during the COVID-19 pandemic was 13.52% (95% CI: 9.06–17.98, *I*^2^ = 65.5%, *p* = 0.008) (Figure 2). The *I*^2^ index showed that there was a high heterogeneity among the studies. Based on the results of the Eger test (*p* = 0.176), publication bias was insignificant in the present study (Figure 3).

**Table 2:** Specifications of studies included in the umbrella review.

**First author**	**Country**	**Sample size**	**Prevalence of PTSD**	**Number of articles**	**Subjects**	**Number of articles related to COVID-19**	**Quality assessment**	**Hetero geneity**
de Pablo GS (20)	UK	470	7.7% (95% CI: 5.6–10.5%)	115	Nurses, Physicians, Medical Students, Social Workers	1	Moderate	*I*^2^ <0.001
Marvaldi M (21)	France	23,432	21.5% (95%CI, 11.2–31.8%),	70	Physicians Nurses	6	Critically low	*I*^2^ = 99.62%
Fan FC (22)	China	17,153	19% (95% CI: 6.4–44.8)	130	Healthcare workers	3	Critically low	*P* = 0.022
Cénat JM (23)	Canada	4,196	21% (95% CI: 0.5–57)	55	Healthcare workers	4	Low	*I*^2^ = 99.72
Batra K (24)	USA	3,676	11.4% (95% CI: 3.6–30.9%)	65	Healthcare workers	6	Moderate	*I*^2^ = 99.2%
Li Y (25)	UK	97,333	21.5% (95% CI: 10.5–34.9%)	65	Healthcare workers	9	Moderate	*I*^2^ = 99.7%
Krishnamoorthy Y (26)	India	171,571	13% (95% CI: 11–16%)	50	Healthcare workers	2	Moderate	NA

*NA, Not Applicable*.

## Discussion

The prevalence of PTSD among HCWs during the COVID-19 pandemic is 13.52%. Previous studies have documented a prevalence of PTSD among HCWs between 21.5% (27) and 38% (28). This can be compared with the prevalence in the general population, ranging between 7% and 53.8% (29). In a study by Busch et al. (30), which evaluated the prevalence of psychological disorders among health care workers during two decades of SARS, H1N1, Ebola, MERS, and Covid 19, a prevalence of 24.51 was reported for PTSD which is consistent with our study. Another study by Wu et al. (31) on mental health disorders in health care workers and the general population revealed that the prevalence of mental health disorders in health care workers is much higher than in the general population These results highlight the negative impact of the COVID-19 pandemic on the mental health of HCWs. Contributing factors unique to the experience of HCWs during the pandemic include prolonged direct contact with patients infected with COVID-19, extended work hours, and an increased workload. Inadequate provisions of personal protective equipment and prolonged wearing of personal protective clothes (covers, shields, face masks, etc.) have fostered an environment of significant physical and mental strain for HCWs. Furthermore, there have been substantial stressors attributable to the occupational hazard of being a HCW during the pandemic. HCWs face the personal risk of contracting and/or transmitting COVID-19 while coping with the loss of many colleagues and dealing with the ethical challenges of clinical decision-making amid a scarcity of resources (25, 32). Previous studies demonstrate that mental illness among HCWs negatively impacts the quality of the health care provided (4, 5). Studies of HCWs experiencing PTSD during the COVID-19 pandemic have identified the following risk factors: female gender, younger age, frontline worker role, career in nursing, and lack of confidence in personal protective equipment (33–35). According to studies of HCWs caring for patients during the SARS epidemic, new cases of psychological disorders, especially severe PTSD, were observed in 5 to 10% of HCWs 4–5 years subsequent to their direct involvement (36–38). It is expected for HCWs to experience a higher incidence of COVID-19-related psychological problems, including PTSD, in future (32, 33). Therefore, hospital leadership should pay special attention to the mental well-being of their employees, especially HCWs, as the mismanagement of their mental health can lead to dire public health consequences. Identifying HCWs at risk factors or the development of psychological disorders, especially PTSD, and providing practical coping strategies are essential to ensuring the quality of the health care workforce.

## Limitations

The limitations of this study included the lack of access to the full texts of some studies, high heterogeneity among the studies, and the lack of reporting heterogeneity, gender, and the specialization of HCWs in some studies.

## Conclusion

The results of this umbrella review show that the overall prevalence of PTSD among HCWs during the COVID-19 pandemic was relatively high. This reality argues strongly for the provision of mental health resources to HCWs during this pandemic. Given the essential role of HCWs, especially during a pandemic, interventions to prevent and address mental illness in this population are paramount. Practical interventions may include holding personal or group counseling sessions, providing education regarding mental illness and prevention strategies, offering mental health counseling, or providing social media outreach resources. Due to the prolongation of this pandemic, health policy makers may consider additional support measures such as efforts to reduce workloads, recruit new staff, provide adequate PPE, offer financial and psychological support, and shorten working hours.

## Data Availability Statement

The datasets presented in this study can be found in online repositories. The names of the repository/repositories and accession number(s) can be found in the article/Supplementary Material.

## Author Contributions

AS, AY, KA, YJ, and MG designed the review, developed the inclusion criteria, screened titles and abstracts, appraised the quality of included papers, and drafted the manuscript. AS, SM, AY, KA, HS, UW, MT, and MG reviewed the study protocol and inclusion criteria and provided substantial input to the manuscript. AS, MG, SM, MT, UW, and KA reviewed the study protocol. AS and MG read and screened articles for inclusion. All authors critically reviewed drafts and approved the final manuscript.

## Conflict of Interest

The authors declare that the research was conducted in the absence of any commercial or financial relationships that could be construed as a potential conflict of interest.

## Publisher's Note

All claims expressed in this article are solely those of the authors and do not necessarily represent those of their affiliated organizations, or those of the publisher, the editors and the reviewers. Any product that may be evaluated in this article, or claim that may be made by its manufacturer, is not guaranteed or endorsed by the publisher.
