# Supplementary material for: The Prevalence of Post-traumatic Stress Disorder Among Health Care Workers During the COVID-19 Pandemic: An Umbrella Review and Meta-Analysis
Source: Front Psychiatry. 2021 Nov 15;12:764738. doi: 10.3389/fpsyt.2021.764738 (PMC8634396; doi:10.3389/fpsyt.2021.764738)
Supplement: Supplementary file 1 [file Table_1.DOCX]

**Appendix 1**

**Table A1**

Using database-appropriate syntax, with parentheses, Boolean operators, and field codes.

| 12 | ((insomnia* OR “Primary Insomnia” OR “Transient Insomnia” OR “Secondary Insomnia” OR “Sleep Initiation Dysfunction*” OR Sleeplessness OR “Insomnia Disorder*” OR “Chronic Insomnia” OR “Psychophysiological Insomnia” OR “Mental health Disorder*” OR “Psychiatric Disorder*”) AND (“2019 novel coronavirus disease” OR COVID19 OR “COVID-19 pandemic” OR “SARS-CoV-2 infection” OR “COVID-19 virus disease” OR “2019 novel coronavirus infection” OR “2019-nCoV infection” OR “Coronavirus disease 2019” OR “2019-nCoV disease” OR “COVID-19 virus infection”) AND (“Health Personnel” OR “Health Care Provider*” OR “Health worker*” OR “Healthcare Provider*” OR “Healthcare Worker*” OR “Health care professional*” OR “medical staff” OR “Medical worker*”) AND (“Systematic review”) AND (“meta-analysis”[tiab] OR “meta-analytic”)) | pubmed |
| --- | --- | --- |
| 77 | ((ALL(insomnia*) OR ALL(“Primary Insomnia”) OR ALL(“Transient Insomnia”) OR ALL(“Secondary Insomnia”) OR ALL(“Sleep Initiation Dysfunction*”) OR ALL(Sleeplessness) OR ALL(“Insomnia Disorder*”) OR ALL(“Chronic Insomnia”) OR ALL(“Psychophysiological Insomnia”) OR ALL(“Mental health Disorder*”) OR ALL(“Psychiatric Disorder*”)) AND (ALL(“2019 novel coronavirus disease”) OR ALL(COVID19) OR ALL(“COVID-19 pandemic”) OR ALL(“SARS-CoV-2 infection”) OR ALL(“COVID-19 virus disease”) OR ALL(“2019 novel coronavirus infection”) OR ALL(“2019-nCoV infection”) OR ALL(“Coronavirus disease 2019”) OR ALL(“2019-nCoV disease”) OR ALL(“COVID-19 virus infection”)) AND (ALL(“Health Personnel”) OR ALL(“Health Care Provider*”) OR ALL(“Health worker*”) OR ALL(“Healthcare Provider*”) OR ALL(“Healthcare Worker*”) OR ALL(“Health care professional*”) OR ALL(“medical staff”) OR ALL(“Medical worker*”)) AND (ALL(“Systematic review”)) AND (TITLE-ABS(“meta-analysis”) OR ALL(“meta-analytic”))) | Scopus |
| 3 | ((TS=(insomnia*) OR TS= (“Primary Insomnia”) OR TS= (“Transient Insomnia”) OR TS= (“Secondary Insomnia”) OR TS= (“Sleep Initiation Dysfunction*”) OR TS= (Sleeplessness) OR TS= (“Insomnia Disorder*”) OR TS= (“Chronic Insomnia”) OR TS= (“Psychophysiological Insomnia”) OR TS= (“Mental health Disorder*”) OR TS= (“Psychiatric Disorder*”)) AND (TS= (“2019 novel coronavirus disease”) OR TS= (COVID19) OR TS= (“COVID-19 pandemic”) OR TS= (“SARS-CoV-2 infection”) OR TS= (“COVID-19 virus disease”) OR TS= (“2019 novel coronavirus infection”) OR TS= (“2019-nCoV infection”) OR TS= (“Coronavirus disease 2019”) OR TS= (“2019-nCoV disease”) OR TS= (“COVID-19 virus infection”)) AND (TS= (“Health Personnel”) OR TS= (“Health Care Provider*”) OR TS= (“Health worker*”) OR TS= (“Healthcare Provider*”) OR TS= (“Healthcare Worker*”) OR TS= (“Health care professional*”) OR TS= (“medical staff”) OR TS= (“Medical worker*”)) AND (TS= (“Systematic review”)) AND (Ti= (“meta-analysis”) OR TS= (“meta-analytic”))) | Web of science |
